# Optimization of Solid-State Fermentation Process for Dietary Fiber in Flaxseed Meal and Analysis of Its Microstructure and Functional Properties

**DOI:** 10.3390/foods14101722

**Published:** 2025-05-13

**Authors:** Chunpeng Hou, Yiyang Zhang, Jiaxun Chen, Jianguo Hu, Chenxian Yang, Fusheng Chen, Tingwei Zhu, Ying Xin, Xiaohui Geng

**Affiliations:** College of Food Science and Engineering, Henan University of Technology, Zhengzhou 450001, China; hcp011016@163.com (C.H.); 13684782360@163.com (Y.Z.); 19233832191@163.com (J.C.); 17324599323@163.com (J.H.); zhutingwei@haut.edu.cn (T.Z.); huilier323@126.com (Y.X.); gengxiaohui970805@163.com (X.G.)

**Keywords:** flaxseed meal, dietary fiber, solid-state fermentation, microstructure, functional properties

## Abstract

Flaxseed meal (FSM) is a by-product of flaxseed product production that is wasted unreasonably at present. In this study, we used *Bacillus subtilis* K6, a dominant microbial strain, for solid-state fermentation (SSF) of FSM following preliminary screening to improve FSM utilization efficiency and enhance the soluble dietary fiber (SDF) content while modifying its functional properties. FSM’s microstructure was characterized before and after fermentation, and the functional properties of the dietary fiber (DF) in the FSM were assessed. Single-factor experiments combined with response surface methodology were conducted to optimize SSF parameters using SDF yield as the response variable. The optimal conditions were determined as follows: 45 h fermentation time, 40.5 °C temperature, and 1:0.65 material-to-liquid ratio. Under these conditions, the SDF yield reached 33.45 ± 0.24%, an SDF yield increase of 36.92%. Scanning electron microscopy and confocal laser scanning microscopy demonstrated FSM’s structural disruption during fermentation. Furthermore, SDF and insoluble DF showed improved water-holding, oil-holding, and swelling capacities following fermentation. These results indicate that SSF effectively enhances the SDF content in FSM and optimizes its functional properties, thereby providing a theoretical foundation for the valorization of flaxseed by-products.

## 1. Introduction

Flaxseed, also called linseed, originated in Central Asia and the Mediterranean region and was later introduced to China, where it has become one of the primary oilseed crops. Its seeds demonstrate two distinct color phenotypes: golden-yellow and reddish-brown [1]. Extensively cultivated in northwestern and northern China, flaxseeds are rich in lipids, proteins, and DF [2]. Flaxseed meal (FSM), a by-product of cold-pressed, non-dehulled flaxseed, retains most of the seed’s nutritional components, including polyunsaturated fatty acids, high-quality proteins, and DF. Its proximate composition closely resembles that of other oilseed meals, such as soybean meals [3,4]. However, FSM’s nutritional profile may considerably differ depending on the cultivar, agroclimatic conditions, oil extraction methods, and processing parameters, resulting in compositional heterogeneity across various FSM sources [5].

DF, defined as carbohydrates resistant to enzymatic digestion in the small intestine, constitutes a crucial component of healthy diets and is recognized as the “seventh nutrient” [6,7]. DF is categorized as soluble (SDF) or insoluble (IDF) based on its aqueous solubility. SDF is especially valued for its superior functional properties, including hydration capacity, viscosity, and fermentability [8]. Furthermore, SDF shows hypoglycemic and hypocholesterolemic effects, promotes the growth of beneficial gut microbiota, and improves host health [9]. SDF shows greater bioactivity and processing adaptability than IDF [10]. Currently, SDF exhibits substantial demand in consumer markets. In food processing, SDF is often added at higher levels than IDF to improve food properties, enhance product stability, and prolong shelf life [11,12]. However, SDF constitutes a minor fraction of natural plant cell walls compared to IDF [13], limiting its application in functional foods. Thus, DF modification is necessary to improve its physicochemical and functional attributes for industrial applications [14].

SSF has emerged as an efficient and eco-friendly strategy for modifying DF [15,16]. During SSF, the microbial consortia enzymatically convert complex polysaccharides into simple bioactive compounds [17,18]. Meng et al. [19] employed *B. subtilis* BSNK-5 for SSF of okara, achieving an SDF yield of 13.14% with enhanced antioxidant, hypoglycemic, and hypolipidemic activities in both SDF and IDF. Cheng et al. [20] utilized mixed microbial consortia to ferment navel orange peel, producing SDF with porous microstructures, elevated antioxidant activity, and improved glucose/cholesterol-binding capacities. Wu et al. [21] demonstrated that *Trichoderma* spp. fermentation of soybean dregs increased SDF content from 4.97 to 18.82%, with the modified SDF mitigating intestinal inflammation and modulating gut microbiota to promote short-chain fatty acid production in murine models. *Bacillus* spp., which are widely used in SSF, secrete carbohydrases, proteases, and lipases that catalyze the degradation of lignocellulosic structures and anti-nutritional factors [22]. Thus, they facilitate IDF bioconversion to SDF. These enzymatic transformations improve the SDF yield and enhance the functional properties [23,24]. Therefore, microbial fermentation represents a promising biotechnological approach to valorize agro-industrial by-products by augmenting their nutritional and functional value [25,26]. Li et al. [27] reported that lactic acid bacteria fermentation elevated the SDF yield in proso millet bran from 4.2 to 7.6%, accompanied by increased aroma and flavor profiles. Similarly, Chen et al. [28] showed that *Trichoderma viride* SSF of tea residues increased the SDF yield from 4.3 to 31.56% under optimized conditions, with concurrent improvements in metal ion-binding capacity. Yang et al. [29] further revealed that the co-fermentation of navel orange peel with *T. viride* and *Aspergillus niger* elevated the SDF yield from 6.63 to 15.73%, along with improved functional properties. Recent advances in microbial biotechnology have facilitated the sustainable conversion of underutilized lignocellulosic by-products into value-added functional and nutritional ingredients.

Preliminary screening of five dominant microbial strains isolated from diverse substrates revealed that *B. subtilis* K6 exhibited superior efficacy in enhancing SDF yield from FSM while demonstrating favorable safety profiles as a probiotic candidate. Consequently, in this study, we aimed to investigate the optimal conditions for improving SDF content in FSM via SSF using *Bacillus subtilis K6* and to systematically analyze the microstructural modifications and functional properties of SDF and IDF before and after fermentation. This study provides novel insights into DF preparation from FSM and their potential application in the development of value-added functional products.

## 2. Materials and Methods

### 2.1. Materials and Reagents

Commercially available flaxseed originating from Inner Mongolia, China; FSM prepared by cold-pressing flaxseeds followed by pulverization, defatted through 3–4 cycles of petroleum ether extraction and air-dried before use; and thermostable α-amylase (4 × 10^4^ U/g) and alkaline protease (2 × 10^5^ U/g) were procured from Solarbio Science & Technology Co., Ltd. (Beijing, China). Luria-Bertani (LB) and De Man, Rogosa, and Sharpe (MRS) broth media were obtained from Beijing Aoboxing Bio-Tech Co., Ltd. (Beijing, China). All other chemicals used in this study were of analytical grade.

Bacterial strains used in this study were *Bacillus subtilis* K6, *Burkholderia* sp. D6, *Cupriavidus* sp. MPD8, *Bacillus subtilis* TB3, and *Lactiplantibacillus plantarum* P34. Specifically, *Burkholderia* sp. D6 and *B. subtilis* TB3 were obtained from soil samples, *B. subtilis* K6 and *Cupriavidus* sp. MPD8 were isolated from compost material, while *L. plantarum* P34 was derived from fermented kimchi. *Bacillus subtilis* K6 (China Center for Type Culture Collection (CCTCC) NO:M 20231096), a strain previously isolated and optimized in our laboratory, was deposited at the CCTCC, Wuhan, China.

### 2.2. Culture Medium Preparation

LB liquid medium (g/L): tryptone (10 g), NaCl (10 g), and yeast extract powder (5 g) were dissolved in deionized water. The pH was adjusted to 7.0, and the solution was autoclaved at 121 °C and 15 psi for 20 min.

The MRS liquid medium was prepared by dissolving MRS broth (49.3 g/L) in deionized water. The solution was sterilized by autoclaving at 121 °C and 15 psi for 20 min. The prepared media were stored at 4 °C for further use.

SSF Medium: FSM (10 g dry weight) was homogenously packed into the fermentation vessels and sterilized by autoclaving at 121 °C and 15 psi for 20 min.

### 2.3. Microbial Cultivation

#### 2.3.1. Seed Culture Preparation

The cryopreserved microbial suspensions (100 μL aliquot) were aseptically inoculated in 40 mL sterile liquid medium (LB broth for *B. subtilis* K6, *Burkholderia* sp. D6, *Cupriavidus* sp. MPD8, and *B. subtilis* TB3; MRS broth for *Lactiplantibacillus plantarum* P34), followed by incubation at 37 °C under agitation (180 rpm) until reaching the mid-exponential growth phase.

#### 2.3.2. Fermentation Broth Preparation

The seed culture was inoculated at 5% (*v*/*v*) into a fresh liquid medium of identical composition and incubated under equivalent conditions (37 °C, 180 rpm) to achieve secondary logarithmic growth, ensuring the optimization of cellular metabolic activity before the fermentation experiments.

### 2.4. Extracting SDF

The fermented and unfermented FSM samples were called F-FSM and U-FSM, respectively. The extraction procedure was conducted as described by Liao et al. [30] with slight modifications. FSM was weighed in a beaker and mixed with distilled water at a ratio of 1:20 (*w*/*v*). The fermentation product’s pH was adjusted to 6.5 ± 0.1, followed by the addition of 1% (*w*/*w*) heat-resistant α-amylase. The mixture was subsequently incubated in a water bath at 95 °C with continuous stirring for 35 min. The pH was then readjusted to 9.5 ± 0.1 and 1% (*w*/*w*) alkaline protease was added, followed by incubation at 65 °C with stirring for 2 h. Following the enzymatic treatment, the mixture was heated in a boiling water bath for 10 min for enzyme inactivation, and the supernatant was collected following centrifugation at 5000× *g* for 10 min. Four volumes of ethanol (95%) were added to the supernatant and the resulting precipitate was collected by centrifugation at 5000× *g* for 10 min. The residual pellet was washed two times with distilled water and mixed with four volumes of ethanol (95%). After standing for 12 h, the precipitate was collected by centrifugation at 5000× *g* for 10 min and freeze-dried to obtain SDF. The SDF and IDF extracted from U-FSM were called CK-SDF and CK-IDF, respectively, whereas those from F-FSM were named F-SDF and F-IDF, respectively.

The SDF yield was calculated using the following formula:

$$\mathrm{SDF}\;\mathrm{yield}\;(\%)=\frac{W_{S}}{W_{M}}\times 100,$$

where W_S_ and W_M_ are the weights of the SDF and FSM, respectively.

### 2.5. Screening of Microbial Strains for the Modification of SDF Fermentation

Different microbial strains were individually inoculated into SSF media and incubated under the following conditions: 37 °C fermentation temperature, 48 h duration, 10% inoculum size, and 1:0.7 substrate-to-liquid ratio. Subsequently, SDF was extracted from the fermented substrates. The dominant strains were selected for further investigation based on the SDF yield.

### 2.6. Optimization of the Fermentation Conditions

#### 2.6.1. Single-Factor Test

The effects of fermentation time (1.5, 2, 2.5, 3, and 3.5 d), temperature (27, 32, 37, 42, and 47 °C), and material-to-liquid ratio (1:0.5, 1:0.6, 1:0.7, 1:0.8, and 1:0.9 g/mL) on SDF yield were separately investigated.

#### 2.6.2. Response Surface Methodology (RSM)

Based on the single-factor test results, three critical parameters (fermentation time, fermentation temperature, and material-to-liquid ratio) that significantly affected SDF yield were selected as independent variables. Optimal ranges were determined using a preliminary single-factor test. A three-factor, three-level Box–Behnken central composite design was implemented with the SDF yield as the response value. Table 1 presents the experimental design matrix showing the factor levels.

### 2.7. Chemical Composition Determination

The moisture, ash, protein, and fat contents of FSM were determined using Chinese National Standards methods. The SDF and IDF contents in the FSM were quantified according to the AOAC Official Method 991.43 [31].

### 2.8. Structural Characterization

#### 2.8.1. Scanning Electron Microscopy (SEM)

The surface morphologies of the FSM samples before and after fermentation were determined using SEM (ZEISS Sigma 360, Oberkochen, Germany). The FSM samples before and after fermentation were dried to a constant weight and adhered to short cylindrical aluminum sheets using a conductive adhesive. The residual powder was gently removed using a dust blower bulb, followed by gold sputter-coating. SEM analysis was subsequently conducted at an accelerating voltage of 10 kV, with observations conducted at various magnifications (500× and 1000×).

#### 2.8.2. Confocal Laser Scanning Microscopy (CLSM)

##### Staining Procedure

Working concentrations of the fluorescent dyes were prepared as follows: calcofluor white (CW, Yuanye, Co., Ltd., Shanghai, China) at 0.1% (*w*/*v*), fluorescein isothiocyanate (FITC, Macklin Co., Ltd., Shanghai, China) at 0.02% (*w*/*v*), and rhodamine B (RhB, Yuanye, Co., Ltd., Shanghai, China) at 0.01% (*w*/*v*). Approximately 10 mg each of U-FSM and F-FSM powdered sample was transferred to a 1.5 mL PCR tube, mixed with 200 μL of the dye cocktail, and incubated at room temperature (25 °C) under light-protected conditions for 30 min. The mixture was centrifuged at 5000× *g* for 3 min to pellet the stained particles. The supernatant was discarded and the residual dye was removed by washing two times with deionized water. Finally, 100 μL of deionized water was added to resuspend the stained sample.

##### Imaging Analysis

A 30 μL aliquot of the stained sample was transferred onto a glass slide using a blunted pipette tip, followed by coverslip placement. The prepared slide was subsequently subjected to imaging analysis under a laser scanning confocal microscope (600× and 400× magnifications with corresponding scale bars of 30 μm and 50 μm, respectively). The fluorescence parameters were as follows: CW (Ex/Em = 405/430 nm, indigo emission), FITC (Ex/Em = 480/517 nm, green emission), and RhB (Ex/Em = 545/575 nm, red emission).

### 2.9. Functional Features

#### 2.9.1. Water-Holding Capacity (WHC)

Samples (0.1 g of CK-SDF, F-SDF, CK-IDF, and F-IDF) were hydrated with 10 mL distilled water and mixed at room temperature (25 °C) for 1 h. The total weight of the mixture in the centrifuge tube system was recorded. Following centrifugation at 10,000× *g* for 10 min, the supernatant was discarded and the combined weight of the precipitate and tube was measured. All procedures were performed in triplicates. WHC was calculated as follows:

$$\mathrm{WHC}\;(g/g)=\frac{m_{2}-m_{1}}{m_{2}},$$

where m_2_ and m_1_ represent the wet and dry sample weights, respectively.

#### 2.9.2. Oil-Holding Capacity (OHC)

Samples (0.1 g of CK-SDF, F-SDF, CK-IDF, and F-IDF) were blended with 10 mL of peanut oil and vortexed at room temperature (25 °C) for 1 h. The total weight of the mixture in the centrifuge tube system was determined. After centrifugation at 10,000× *g* for 10 min, the supernatant was removed and the precipitate tube assembly was weighed. All measurements were performed in triplicates. The OHC was calculated as follows:

$$\mathrm{OHC}\;(g/g)=\frac{m_{2}-m_{1}}{m_{2}},$$

where m_2_ and m_1_ represent the wet and dry sample weights, respectively.

#### 2.9.3. Swelling Capacity (SC)

The sample (CK-SDF, F-SDF, CK-IDF, or F-IDF) was accurately weighed (0.5 g) and transferred to a graduated cylinder containing 10 mL of distilled water. The initial volume (V_1_) was recorded. After vortex homogenization, the mixture was maintained at ambient temperature (25 °C) for 24 h. Subsequently, the final swollen volume of the DF (V_2_) was measured. The SC was calculated using the following equation:

$$\mathrm{SC}\;(\mathrm{mL}/g)=\frac{V_{2}-V_{1}}{m},$$

where m represents the dry sample weight.

### 2.10. Statistical Analyses

The results of the experiments are provided as the mean ± standard deviation (*n* ≥ 3). The response surface was designed using DesignExpert version 13.0. Statistical analysis and chart mapping were completed using IBM SPSS Statistics 27.0 Origin 2024 and PowerPoint. The differences between means were assessed by analysis of variance and Duncan’s test, using a significance level of *p* < 0.05.

## 3. Results and Discussion

### 3.1. Screening of Microbial Strains for SDF Fermentation Modification

**Figure 1:**
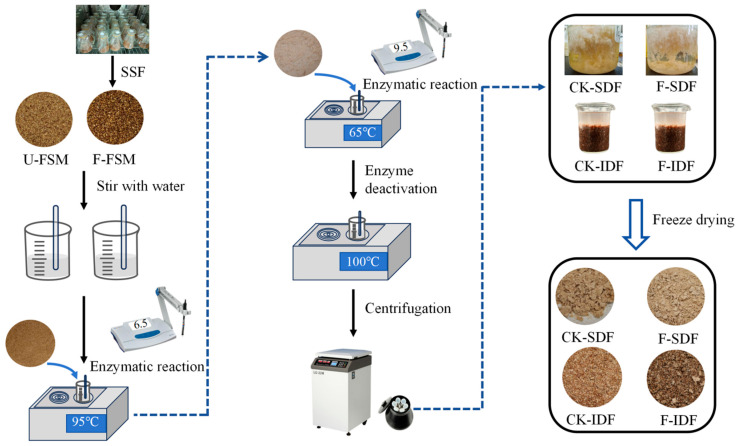
Flowchart of dietary fiber extraction process from unfermented (U-FSM) and fermented flaxseed meal (F-FSM).

**Figure 2:**
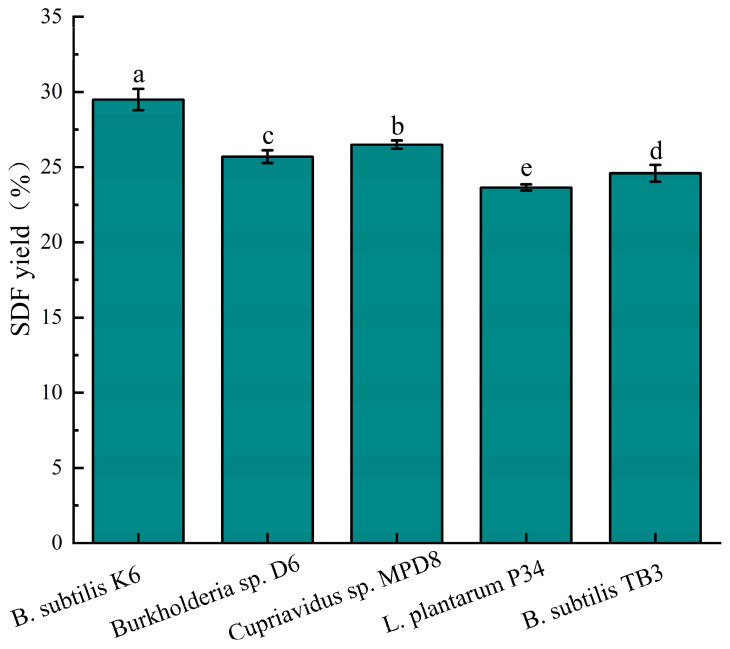
Effects of different microbial strains on the yield of soluble dietary fiber. A significant difference between samples is indicated by different lowercase letters (*p* < 0.05).

Figure 1 illustrates the process flowchart of DF extraction from the U-FSM and F-FSM. The SDF yields obtained from five distinct microbial strains under identical fermentation conditions were compared (Figure 2). Notably, the *B. subtilis* K6 strain showed the highest F-SDF content of 29.5 ± 0.7%, which was considerably higher than the other four strains. Consequently, *B. subtilis* K6 was selected as the experimental strain for subsequent fermentation studies.

### 3.2. Single-Factor Test

**Figure 3:**
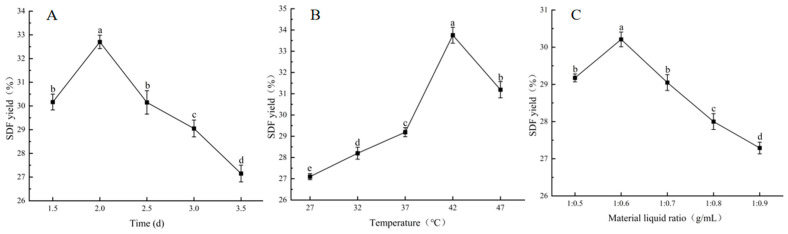
Effect of different variables on the yield of soluble dietary fiber. (**A**) The fermentation time, (**B**) fermentation temperature, (**C**) material to liquid ratio. A significant difference between samples is indicated by different lowercase letters (*p* < 0.05).

Figure 3 illustrates the effects of fermentation time, temperature, and material-to-liquid ratio on SDF yield from FSM. The SDF yield peaked following 2 days of fermentation. During this process, *B. subtilis* K6 progressively degraded the IDF components in FSM, releasing and converting them into SDF. The limited IDF decomposition during the initial fermentation stages led to lower SDF yields. Contrastingly, prolonged fermentation resulted in microbial utilization of SDF components, which were subsequently converted into low-molecular-weight sugars that escaped ethanol precipitation [32], consequently reducing SDF recovery.

Maximum SDF yield occurred at 42 °C fermentation temperature. Suboptimal temperatures inhibit microbial proliferation and metabolic activity. Conversely, increased temperatures accelerate the evaporation of moisture from the culture medium. This creates hyperosmotic stress and potentially denatures thermolabile enzymes, both of which ultimately impair SDF biosynthesis.

The optimal material-to-liquid ratio for SDF production was identified as 1:0.6 (g/mL). Insufficient hydration restricts microbial growth kinetics, whereas excessive moisture induces substrate agglomeration and viscosity elevation. This ultimately compromises the mass transfer efficiency and metabolic activity, which are critical parameters governing SDF yield.

In summary, different fermentation conditions markedly influence the growth rate and metabolic activities of microorganisms and the enzyme stability and kinetic properties [33]. Moreover, the individual enzymes demonstrate distinct optimal pH and temperature activity profiles. Deviations from these critical ranges may compromise catalytic efficiency via structural denaturation or altered substrate affinity [34]. The intricate interplay between *B. subtilis* K6-mediated IDF conversion to SDF remains mechanistically undefined, owing to the complexity of SSF systems. Subsequent studies will employ enzymatic activity assays (e.g., cellulases, hemicellulases) integrated with multi-omic analyses to systematically decode the catalytic pathways driving IDF depolymerization and SDF yield optimization.

### 3.3. Optimization of the Fermentation Conditions

Based on the single-factor experimental findings, three critical parameters (fermentation time, temperature, and solid–liquid ratio) were selected for Box–Behnken RSM optimization, with SDF yield as the response variable. Table 1 lists the experimental parameters and corresponding results. The 17 different experimental combinations of the response surface are shown in Table 2.

The experimental data were fitted to a quadratic polynomial regression model using the Design Expert 13. The derived second-order regression equation for fermentation time (A), temperature (B), and solid–liquid ratio (C) was Y = 34.28 − 0.77A − 0.7638B + 1.86C − 0.0475AB + 0.2275AC + 0.4850BC − 1.93A^2^ − 0.8756B^2^ − 1.73C^2^.

Analysis of variance showed that the regression model showed exceptional significance (*p* < 0.01) with a non-significant lack-of-fit term (*p* = 0.3347 > 0.05), confirming model adequacy (Table 3). The high determination coefficient (R^2^ = 0.9746) and adjusted R^2^ value (R_Adj_^2^ = 0.9419) showed an excellent predictive capability for SDF yield optimization. The first-order terms (A, B, and C) and quadratic terms (A^2^, B^2^, and C^2^) all revealed significant effects (*p* < 0.05). Comparative F-values showed the following parameter influence hierarchy: solid–liquid ratio (C) > fermentation time (A) > temperature (B).

**Figure 4:**
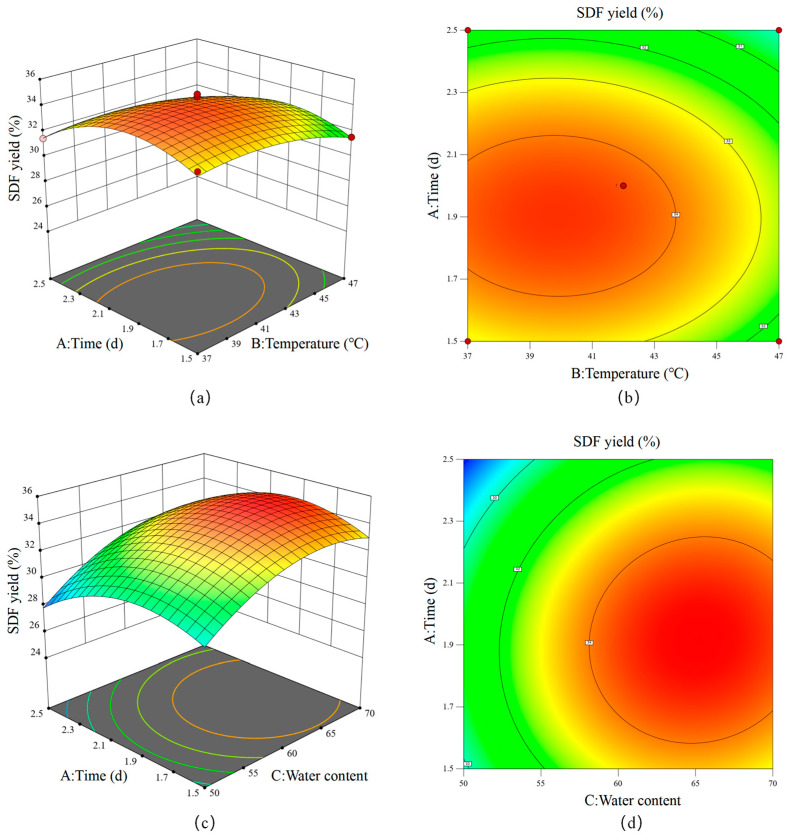

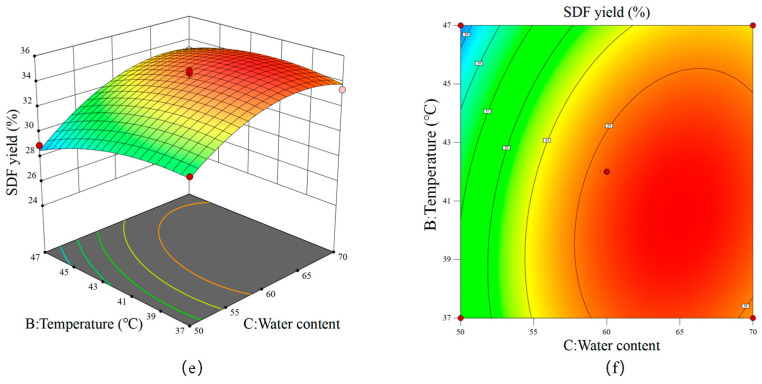
Response surface plots. The effect of time and temperature on the yield of soluble dietary fiber (SDF) (**a**,**b**). The effect of time and water content on the SDF yield (**c**,**d**). The effect of temperature and water content on the SDF yield (**e**,**f**).

As a key component of the regression equation, the three-dimensional response graph (Figure 4) could illustrate the interaction between variables and determine the superlative situations for each variable. Numerical optimization identified optimal conditions for SDF production via SSF with *B. subtilis* K6: fermentation time: 1.88 d, fermentation temperature: 40.39 °C, and material-to-liquid ratio: 1:0.65 (g/mL). The maximum predicted SDF yield under these conditions was 34.89%. Practical validation using adjusted parameters (45 h fermentation, 40.5 °C, 1:0.65 ratio) yielded 33.45 ± 0.24% SDF, showing excellent agreement with model predictions (<5% relative error). This finding confirmed the reliability of the model for industrial process scaling.

### 3.4. Principal Component Analysis

Table 4 presents the proximate compositions of U-FSM and F-FSM. U-FSM contained 39.37 ± 0.52% crude protein, 40.77 ± 0.2% IDF, 24.43 ± 0.7% SDF, along with minor constituents, including lipids, ash, and moisture. These analytical results show that FSM is a valuable source of high-quality protein and dietary fiber components. Notably, the IDF constituted the predominant fraction in the U-FSM. Although SDF and IDF demonstrate distinct functional properties, emerging evidence suggests that SDF possesses superior nutritional significance compared to IDF in human physiology [35]. Post-fermentation analysis revealed a significant increase in protein content in F-FSM, which is likely attributable to microbial-mediated depletion of organic matter. The reduction in the total substrate mass during fermentation led to a concentration effect, resulting in proportional enrichment of residual nitrogenous compounds (proteins) and inorganic ash constituents. This finding aligns with observations in other fermentation systems—*Aspergillus oryzae* SSF peanut meal showed analogous protein enrichment [36], whereas mixed culture fermentation of okara similarly increased protein retention [37].

The most pronounced change was noted in the SDF content of F-FSM after SSF. These results highlight the significance of the SSF-mediated bioconversion of IDF to SDF within flaxseed meal matrices, coupled with the requirement to expand SDF utilization in functional food systems.

### 3.5. Analysis of the Structural Properties

#### 3.5.1. SEM

**Figure 5:**
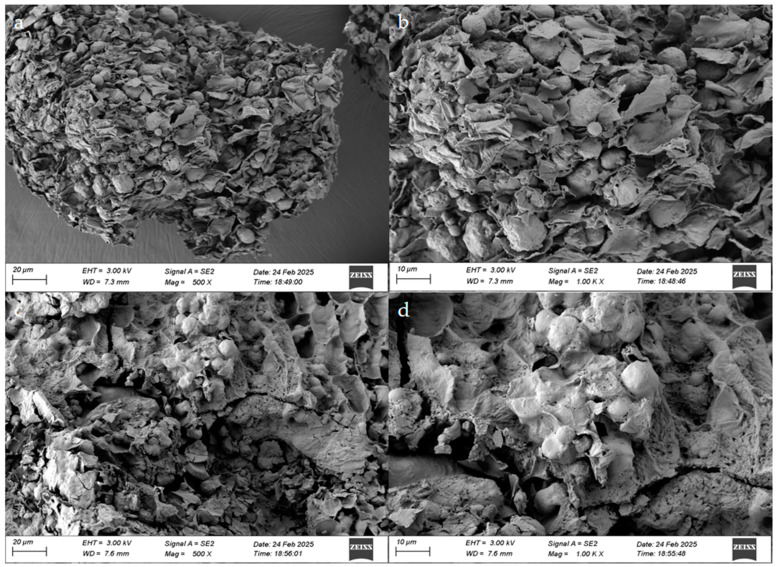
Microscopic topography of flaxseed meal (FSM) before and after fermentation: unfermented flaxseed meal (U-FSM) (**a**,**b**), fermented flaxseed meal (F-FSM) (**c**,**d**). (Magnification: 500× **a**,**c**; 1000× **b**,**d**).

SEM showed marked morphological changes in the FSM matrices before and after fermentation (Figure 5). The U-FSM showed irregular block-like structures with low porosity, characterized by rough surfaces containing lamellar formations and embedded spheroidal/globular particulates. These surface-adherent deposits, potentially corresponding to starch granules and protein aggregates, were frequently observed superficially and within the internal matrices.

Contrastingly, the F-FSM showed substantial structural disintegration, featuring pronounced surface collapse with microbial-induced erosion patterns. The fermentation process generated microporous structures via partial degradation of the lamellar components. Furthermore, substantial reductions in block-like and spheroidal particulates were noted following fermentation, suggesting the effective bioconversion of macromolecular constituents.

#### 3.5.2. CLSM

**Figure 6:**
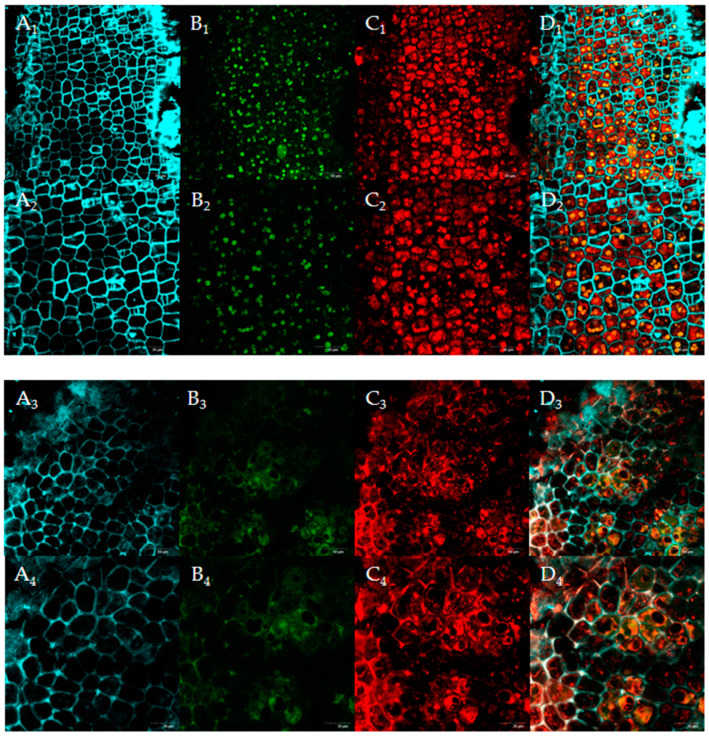
Confocal laser scanning microscopy (CLSM) images of flaxseed meal (FSM) before and after fermentation: unfermented FSM (U-FSM) and fermented FSM (F-FSM) (400× and 600×). (**A_1_**–**A_4_**) fiber (CW), blue; (**B_1_**–**B_4_**) starch (FITC), green; (**C_1_**–**C_4_**) proteins (Rhb), red; (**D_1_**–**D_4_**) merged image. 1: U-FSM 400×, 2: U-FSM 600×, 3: F-FSM 400×, 4: F-FSM 600×.

CLSM characterization showed the composite nature of the FSM, comprising cellulose, protein, and starch matrices (Figure 6). Cellulose, a linear polymer of glucose units linked by β-(1→4)-glycosidic bonds, constitutes the primary structural component of plant cell walls, providing mechanical rigidity and tensile strength [38]. U-FSM showed an intact cellulose network encapsulating the protein and starch components within its fibrous architecture. After SSF, F-FSM showed substantial disintegration of the cellulose superstructure.

Microbial degradation of cellulose, a principal cell wall polysaccharide, into oligosaccharides facilitated the release of entrapped macromolecules. This structural modification aligns with established mechanisms, in which enhanced SDF content is directly correlated with the depolymerization of cell wall polysaccharides during microbial processing [37].

### 3.6. Functional Features

#### 3.6.1. WHC

Microbial fermentation substantially enhanced the WHC of the F-SDF and F-IDF fractions compared to their unmodified counterparts (Table 5). Among the four sample groups, F-SDF exhibited the highest WHC value of 14.49 ± 0.24 g/g. This improvement likely stems from the exposure of hydrophilic moieties during fermentation-mediated structural reorganization [39]. The superior WHC of the modified SDF indicates its potential as a functional hydrocolloid for food applications. Its improved water retention capacity can effectively mitigate dehydration and preserve product quality parameters, including moisture equilibrium and textural characteristics.

#### 3.6.2. OHC

The oil retention capability of DF plays a crucial role in food processing applications. Improved OHC aids in lipid retention during manufacturing while potentially decreasing the levels of serum cholesterol via intestinal oil absorption [40]. Microbial fermentation considerably improved the OHC values of F-SDF and F-IDF (Table 5). Among the four sample groups, F-SDF demonstrated the highest OHC of 9.65 ± 0.09 g/g. This enhancement likely originates from the fermentation-induced structural reorganization of cellulose matrices, which increases the surface area and pore accessibility, which are key determinants of oil adsorption capacity [41]. Additionally, the exposure of specific lipophilic functional groups and altered surface charge density within the modified porous architecture synergistically enhance lipid retention.

#### 3.6.3. SC

The SC of the DF, defined as its ability to increase in volume upon hydration, directly correlates with gastric distension-induced satiety. Higher SC values amplify the occupied gastric space, thereby elevating satiety signals [42]. Post-hydration volumetric expansion stimulates intestinal peristalsis, facilitates fecal bulk formation, and alleviates constipation. Fermentation markedly elevated the SC of F-SDF and F-IDF, with quantified values of 5.0 ± 0.13 mL/g and 1.33 ± 0.24 mL/g, respectively (Table 5). Mechanistically, the fermentation process induces structural modifications in SDF and IDF, including looser matrix architectures and altered surface properties, which collectively contribute to improved water retention and SC [43].

Collectively, these data indicate that the functional properties of F-SDF and F-IDF extracted from *B. subtilis* K6-fermented FSM were improved. Comparative analyses highlight the superior efficacy of *B. subtilis* K6 in FSM fermentation relative to previously reported microbial strains. Ban et al. [24] utilized *Hericium erinaceus* for SSF of corn husk, yet the resultant SDF exhibited inferior WHC (3.65 ± 0.12 g/g), OHC (3.93 ± 0.09 g/g), and SC (4.31 ± 0.04 mL/g) compared to SDF derived from *B. subtilis* K6-fermented FSM in this work. Xu et al. [37] achieved higher WHC, OHC, and SC in SSF-treated soybean residue using mixed microbial consortia; however, the SDF yield (3.62%) was notably lower than our optimized yield. Wang et al. [44] reported an SDF yield of 18.45 ± 0.2% from *B. paracaetocasei* KLDS-82-fermented FSM, which represents a reduction of approximately 45% compared to the yields observed in the present study. Furthermore, *B. subtilis* K6-modified SDF demonstrated superior functional properties. These findings underscore the unique efficacy of *B. subtilis* K6 in balancing high SDF yield with enhanced functionality, positioning it as a superior candidate for valorizing flaxseed by-products.

## 4. Conclusions

Herein, the SSF of FSM using *B. subtilis* K6 was systematically optimized using RSM, leading to a considerable enhancement of the SDF content. The optimal conditions were determined as follows: 45 h fermentation time, 40.5 °C temperature, and 1:0.65 material-to-liquid ratio. Under these conditions, the SDF yield reached 33.45 ± 0.24%, an SDF yield increase of 36.92%. The structural characterization of U-FSM and F-FSM showed considerable microbial-induced modifications, including surface erosion and distinct microstructural changes. CLSM analysis showed that *B. subtilis* K6 disrupted U-FSM’s surface cellulose network, aiding the release of encapsulated polysaccharides and starch granules. These structural reorganizations contributed to enhanced functional properties and nutritional accessibility of the fermented matrix. Subsequent SDF and IDF extraction from U-FSM and F-FSM showed that F-SDF and F-IDF exhibited substantially higher WHC, OHC, and SC compared to the unfermented controls. Notably, F-SDF outperformed F-IDF in WHC (14.49 ± 0.24 g/g), OHC (9.65 ± 0.09 g/g), and SC (5.0 ± 0.13 mL/g), underscoring its superior functionality. Collectively, *B. subtilis* K6-mediated SSF represents an innovative strategy for valorizing FSM by-products. This approach enhances SDF yield and quality. Additionally, it enhances the utilization efficiency and industrial application potential of FSM in food systems, providing dual benefits for sustainably managing resources and developing functional ingredients. Applying modified SDF to food processing can endow food with good sensory characteristics and nutritional value. In summary, the results of this study provided new insights into the comprehensive utilization of agricultural by-products and their potential applications in food industry.

## Figures and Tables

**Table 1:** Factors and levels in the response surface analysis.

Factors	Levels		
	−1	0	1
A—Fermentation time (d)	1.5	2	2.5
B—Fermentation temperature (°C)	37	42	47
C—Material-to-liquid ratio (g/mL)	1:0.5	1:0.6	1:0.7

**Table 2:** Three-factor, three-level Box–Behnken design used for response surface methodology and experimental data of the investigated response.

Run	A	B	C	Y (%)
1	1.5	47	60	31.56 ± 0.11
2	2.5	42	50	27.67 ± 0.06
3	2	47	70	33.12 ± 0.17
4	2	37	70	33.39 ± 0.20
5	2	42	60	34.87 ± 0.05
6	1.5	42	70	33.10 ± 0.22
7	2	42	60	33.79 ± 0.13
8	2.5	47	60	29.56 ± 0.35
9	2	37	50	31.19 ± 0.27
10	2.5	42	70	32.38 ± 0.21
11	2	42	60	34.66 ± 0.08
12	2	47	50	28.98 ± 0.16
13	1.5	37	60	33.28 ± 0.15
14	1.5	42	50	29.30 ± 0.31
15	2.5	37	60	31.47 ± 0.15
16	2	42	60	34.16 ± 0.07
17	2	42	60	33.91 ± 0.22

**Table 3:** Analysis of variance for the response surface quadratic model.

Source	Sum of Squares	Degrees of Freedom	Mean Square	F-Value	*p*-Value	Significance ^a^
Model	73.01	9	8.11	29.82	<0.0001	**
A—Time	4.74	1	7.47	17.44	0.0042	**
B—Temperature	4.67	1	4.67	17.15	0.0043	**
C—Material-to-liquid ratio	27.51	1	27.57	101.33	<0.0001	**
AB	0.0090	1	0.0090	0.0332	0.8606	
AC	0.2070	1	0.2070	0.7610	0.4119	
BC	0.9409	1	0.9409	3.46	0.1052	
A^2^	15.75	1	15.75	57.89	0.0001	**
B^2^	3.23	1	3.23	11.89	0.0107	*
C^2^	12.62	1	12.62	46.40	0.0003	**
Resdual	1.90	7	0.2720			
Lack of fit	1.02	3	0.3401	1.54	0.3347	Not significant
Pure error	0.8839	4	0.2210			
Cor total	74.92	16				
R-Squared	0.9746					
Adj.R-squared	0.9419					
Adeq.precision	16.2970					
C.V. %	1.62					

Note: Significance ^a^: * *p* < 0.05; ** *p* < 0.01.

**Table 4:** Basic composition and content of unfermented (U-FSM) and fermented flaxseed meal (F-FSM).

	Protein (%)	Ash (%)	Fat (%)	Moisture (%)	SDF (%)	IDF (%)
U-FSM	39.37 ± 0.52	4.29 ± 0.1	4.75 ± 0.25	5.52 ± 0.2	24.43 ± 0.7	40.77 ± 0.2
F-FSM	46.51 ± 0.27	4.74 ± 0.15	2.5 ± 0.5	3.18 ± 0.14	33.45 ± 0.24	28.27 ± 0.13

**Table 5:** WHC, OHC, and SC of SDF and IDF before and after fermentation.

Item	CK-SDF	F-SDF	CK-IDF	F-IDF
WHC	12.3 ± 0.14 ^b^	14.49 ± 0.24 ^a^	10.91 ± 0.2 ^c^	12.26 ± 0.21 ^b^
OHC	5.97 ± 0.1 ^c^	9.65 ± 0.09 ^a^	4.34 ± 0.11 ^d^	7.15 ± 0.13 ^b^
SC	4.3 ± 0.21 ^b^	5.0 ± 0.13 ^a^	0.67 ± 0.17 ^d^	1.33 ± 0.24 ^c^

Data are expressed as mean ± standard deviation (*n* = 3). Different letters in the same column indicate significant differences (*p* < 0.05). The same letters indicate non-significant differences (*p* > 0.05). CK: control group; F: fermentation group; SDF: soluble dietary fiber; IDF: insoluble dietary fiber; WHC, water-holding capacity; OHC, oil-holding capacity; SC, swelling capacity.

## Data Availability

The original contributions presented in the study are included in the article, further inquiries can be directed to the corresponding authors.
